# Zinc Oxide Nanoparticles Improve *Pleioblastus pygmaeus* Plant Tolerance to Arsenic and Mercury by Stimulating Antioxidant Defense and Reducing the Metal Accumulation and Translocation

**DOI:** 10.3389/fpls.2022.841501

**Published:** 2022-02-28

**Authors:** Abolghassem Emamverdian, Mirza Hasanuzzaman, Yulong Ding, James Barker, Farzad Mokhberdoran, Guohua Liu

**Affiliations:** ^1^Co-innovation Center for Sustainable Forestry in Southern China, Nanjing Forestry University, Nanjing, China; ^2^Bamboo Research Institute, Nanjing Forestry University, Nanjing, China; ^3^Department of Agronomy, Faculty of Agriculture, Sher-e-Bangla Agricultural University, Dhaka, Bangladesh; ^4^School of Life Sciences, Pharmacy and Chemistry, Kingston University, Kingston-Upon-Thames, United Kingdom

**Keywords:** metal/metalloid toxicity, nanomaterials, tolerance index, ROS metabolism, translocation factor

## Abstract

The utilization of nanoparticles to potentially reduce toxicity from metals/metalloids in plants has increased in recent years, which can help them to achieve tolerance under the stressful conditions. An *in vitro* experiment was conducted to investigate five different levels of zinc oxide nanoparticles (ZnO-NPs; 0, 50, 100, 150, and 200 μM) both alone and in combination with 150 μM arsenic (As) and 150 μM mercury (Hg) in one-year-old *Pleioblastus pygmaeus (Miq.) Nakai* plants through four replications. The results demonstrated that As and Hg alone had damaging effects on the plant growth and development. However, the addition of various concentrations of ZnO-NPs led to increased antioxidant activity, proline (79%) content, glycine betaine (71%) content, tyrosine ammonia-lyase (43%) activity, phenylalanine ammonia-lyase (69%) activity, chlorophyll indices, and eventually plant biomass, while the lipoxygenase activity, electrolyte leakage, soluble protein, hydrogen peroxide content, and thiobarbituric acid reactive substances were reduced. We concluded that ZnO-NPs detoxified As and Hg toxicity in the plants through increasing antioxidant activity, reducing As and Hg accumulation, As and Hg translocation from roots to shoots, and adjusting stomatal closure. This detoxification was further confirmed by the reduction of the translocation factor of As and Hg and the enhancement of the tolerance index in combination with ZnO-NPs. However, there is a need for further investigation with different metals/metalloids.

## Introduction

In recent decades, with the unprecedented acceleration in industrialization and urbanization, contamination from heavy metals (HMs) and metalloids constitutes a considerable health hazard to humans around the world, tainting soil, air, and drinking water (Schlutow et al., 2021). Environmental contamination arising from HM has become an increasingly major dilemma, influencing the atmosphere, water, and soil (Hu et al., 2014). Among these contaminants, arsenic (As) and mercury (Hg) are known to be two of the five key toxic metal/metalloid pollutants (copper, Cu; cadmium, Cd; lead, Pb, As, and Hg) in China (Hu et al., 2014). Today, As is responsible for the pollution of a large volume of groundwater in many other countries (Naidu et al., 2006). According to World Health Organization (WHO) reports, the optimum As in drinking water is 10 μg L^−1^, and they caution that 200 million people worldwide are exposed to the dangers of As toxicity (Abbas et al., 2018). The intensive mining, burning of fossil fuels, metal smelting, and the profligate use of herbicides, insecticides, as well as pesticides are the main sources of As in water, soil, and air (Awasthi et al., 2017). In plants, As leads to the production of oxidative stress with the generation of reactive oxygen species (ROS) components, such as hydrogen peroxide (H_2_O_2_), superoxide radicals (O_2_^•−^), and hydroxyl radicals (•OH; Shahid et al., 2017). This generation of ROS occurs through the conversion of As (V) to As (III; Armendariz et al., 2016). Arsenic in plants leads to a reduction in their morphology, physiology, and growth process. Morphologically, As causes leaf senescence and necrosis, as well as defoliation, leading to a reduction in leaf number and chlorosis. Physiologically, As impacts antioxidant activity, photosynthesis inhibition, and stomatal conductance leading to ROS generation, which can cause DNA damage, lipid peroxidation, carbohydrate damage, and compromised chloroplast membrane. Regarding plant growth, As limits root extension and proliferation, as well as plant biomass, and reduces plant yield (Abbas et al., 2018). Mercury, as one of the natural components of the Earth’s crust, pollutes a large area of land, including many agricultural regions (Montero-Palmero et al., 2014). The main anthropogenic activities that cause toxic release of Hg to the environment include; silver, gold, and Hg mining; fossil fuel combustion; and smelting of non-ferrous metals (Li et al., 2020; Arregui et al., 2021). It is reported that Hg causes contamination of the lands and irrigation water that produce approximately 100,000 tons of crops annually (Sengar et al., 2010). China is the largest anthropogenic source of Hg, with emissions of 500–1,000 t annually (Streets et al., 2005; Obrist et al., 2018). Mercury-stressed plants tend to have high levels of ROS, which induces oxidative stress, leading to cell membrane damage, disruption of membrane permeability, and eventually antioxidant enzymatic and nonenzymatic activation in the plants (Cargnelutti et al., 2006).

Nanoparticles with minute sizes of 1–100 nm can play a unique role in plant biotechnology and toxicology (Cele, 2020). Many studies have explored the prominent role of nanoparticles on nucleotides, plant chemicals, and proteins in various sites within plants (Rastogi et al., 2019). Nanoremediation is an emerging technology used to quickly clean up the environment, and it can be employed as an eco-friendly and environmentally safe material (Sanaeimehr et al., 2018). Among nanoparticles, the economic rationale for the nanoremediation use of zinc oxide nanoparticles (ZnO-NPs) is noteworthy, as they are shown to be more cost-effective than other nanoparticles such as titanium dioxide nanoparticles (TiO_2_ NPs; Raja et al., 2018). ZnO-NPs, because of their chemical catalytic properties and strong physical adsorption, can be a good choice for use in environmental remediation (Jing et al., 2001). Bulk ZnO, due to its low reactivity and solubility, is absorbed with low efficiency by plants. However, ZnO-NPs with tiny size, high rate of dissolution, and optimal specific surface area enhance zinc (Zn) absorption by plants. As a result, they can solve the Zn deficiency problem in plants (Rastogi et al., 2019). Zinc is an essential element in plant metabolism and growth. It is involved in many processes, such as the biosynthesis of enzymes, proteins, and chlorophyll (Chl, Singh et al., 2018), for instance, Zn can reduce oxidative stress in plants by role-playing in chloroplastic and cytosolic Cu/Zn-SOD enzymes (Barker and Pilbeam, 2015). Or it is reported that metalloproteins of Zn are responsible for the regulation of gene expression in plants under oxidative stress (Barker and Pilbeam, 2015). The positive role of ZnO-NPs in the growth and development of plants is reported in many studies (Rizwan et al., 2019b; Adrees et al., 2021; Faizan et al., 2021a,b). It is reported that ZnO-NPs increase the production of secondary metabolites like phenolics in *Melissa officinalis* (Babajani et al., 2019). ZnO-NPs reduce the negative impact of ROS compounds in HM exposed plants by enhancing antioxidant capacity of plants (Ahmad et al., 2020; Faizan et al., 2021b), which is one of the main mechanisms in the amelioration of HMs by ZnO-NPs. However, there are few studies detailing the positive role of ZnO-NPs in decreasing HM toxicity in plants. This study can help to promote bamboo safety when used as a nutrient and economic source for regional populations in China and can introduce bamboo cultivation for use in phytoremediation technology, eventually increasing our understanding of how the mechanisms of ZnO-NPs are involved in the face of metal stress.

Bamboo (*Bambusoideae*) plants are one of the fastest-growing plants with high biomass, and they cover a remarkable area of Chinese forestland (Yao et al., 2020). It is reported that bamboo species cover 31.5 million ha of the world’s forestland (Huang et al., 2020a). In China, bamboo species are classified into 48 genera and 500 species (Huang et al., 2016). Bamboo is an important economic source for the livelihood of the local people in southern and southwestern China (Hogarth and Belcher, 2013). Additionally, bamboo shoots because of their high dietary fiber, high-calorie contents, and low fat are consumed as a tasty nutrient-rich food source by local people in Asia and around the globe (Bal et al., 2012; Emamverdian et al., 2020b). Bamboo species can be particularly used in phytoremediation technology because of their fast vegetative growth and high biomass yields coupled with high metals ions extraction capacities in the roots (Bian et al., 2019). Very few hyperaccumulators possess such features all together. Ornamental plant species are shown to be tolerant to metal stress in urban areas. Therefore, they can be used in remediation schemes as they exhibit phytoremediation potentials (González-Chávez Mdel and Carrillo-González, 2013). Hence, in addition to the commercial purposes (beautification/gardening), they can be used as sustainable and eco-friendly solutions for cleaning up air pollution and removing sewage contamination in urban areas (Liu et al., 2008). *Pleioblastus pygmaeus* is known as an evergreen bamboo species with an average height of 30 cm–50 cm and has often been used for landscape purposes. It was introduced from Japan to China in the early 20th century. It has high tolerance to grow in basic (alkaline), acidic, and neutral soils throughout the year (Huang et al., 2020b) and is cultivated in many areas and provinces in China such as Jiangsu province. On the other hand, HMs are one of the important toxic factors in water and soil in the Southwest regions of China (Emamverdian et al., 2021a,b), which pose significant risks to human health. An early survey conducted on edible plants in some local markets in China showed that the content of As was remarkably high in the shoots of bamboo species (Zhao et al., 2006). On the other hand, it was recently reported that there are 4.1 × 104 metric tons of Hg in agricultural and forestry lands of China (Zhou et al., 2018). Therefore, these two particular metal/metalloids pose a significant threat to human health through the widespread cultivation and consumption of bamboo in China. Hence, it is essential to identify bio-nutrient factors for the removal or reduction of toxicity from the surrounding environment, agricultural, and forest lands. The study aims to improve bamboo plant tolerance under As and Hg toxicity by the application of different concentrations of ZnO-NPs and to pinpoint the mechanisms involved that allow ZnO-NPs detoxification. To our knowledge, this is the first study to investigate the role of ZnO-NPs on bamboo species under the influence of two important toxic metals/metalloids (As and Hg). This can be an important step in the application of ZnO-NPs in plants and environmental detoxification. We hypothesized that the exogenous application of ZnO-NPs can increase bamboo plant tolerance by reducing metal accumulation and the translocation of metals from roots to shoots, stimulating antioxidant activities and promoting closer stomatal regulation.

## Materials and Methods

### Plant Material and Growth Conditions

Single clone 1-year-old branches of *P. pygmaeus* were selected as plant materials, which have been growing since 1982 at Nanjing Forestry University, Bamboo Garden (Nanjing, Jiangsu, China). For shoot production and shoot expansion, long nodal (10 mm) explants were grown under tissue culture conditions conducted in medium of Murashige and Skoog (1962) containing 0.5 ml, 4 ml, 7–10 g L^−1^, and 30 g L^−1^ kinetin, 6-benzyl amino purine, agar, and sucrose. In this regard, roots proliferated from young shoots that were used in glass petri dishes with a constant diameter (60 mm) to keep the MS medium containing 4 μM nicotinic acid, 0.6 mM myoinositol, 1.2 μM thiamine-HCl, 30 g L^−1^ sucrose, 3 μM pyridoxine, 7–10 g L^−1^ agar, and 1 mg L^−1^ Indole-3-acetic acid (IAA) as growth hormones.

In this study, five concentrations of ZnO-NPs (0, 50, 100, 150, and 200 μM) alone or in a combination with two types of toxic metals/metalloids (150 μM As and 150 μM Hg) were used in a completely randomized design (CRD) through four replications. The total duration of the experiment was 3 weeks. Thus, different treatments including ZnO-NPs and As/Hg in a form of powder were added to 1 L MS medium with 30 g of sucrose. The As was prepared from sodium arsenate heptahydrate powder (Na_2_HAsO_4_.7H_2_O) and the Hg was from the salt of white Hg powder (HgCl_2_). After the regulation of the MS medium in an appropriate pH (5.8 ± 0.1), 7–10 g L^−1^ agar was added. Then, the MS medium was transferred to a microwave oven (China Energy Label) for 30 min at an optimum temperature of 120°C. Sterilization of the MS medium was conducted by an autoclave (HiClave HVE-50; ZEALWAY). For the bamboo plantation, an inoculation hood (Air Tech), fluorescent white lamps, and UV light (wavelengths of 10–400 nm) were used at temperatures of 15 and 30°C. Finally, the planted bamboos were preserved in a special chamber room under controlled conditions for 3 weeks.

ZnO-NPs were provided by the Nanjing Jiancheng Company, Nanjing, Jiangsu Province, China. This material has the characteristics of a powder with high nano Zn purity and a diameter of less than 50 nm. In this study, the levels of ZnO-NPs and metals were selected according to the different ranges of bamboo tolerance obtained by our previous studies (Emamverdian et al., 2020a, 2021a,b).

At the end of the experiment, all bamboo samples were separated from the cleaned MS medium. For this study, antioxidant enzyme activities, thiobarbituric acid reactive substances (TBARS), H_2_O_2_ content, soluble protein (SP), proline (Pro), and glycine betaine (GB) content, total phenolics, flavonols, tocopherols, tyrosine ammonia-lyase (TAL) activity and phenylalanine ammonia-lyase (PAL) activity, electrolyte leakage (EL), lipoxygenase activity (LOX), Chl, carotenoid contents, ZnO-NPs, and metal accumulation in plant shoots, stems, and roots were measured carefully. The translocation factor (TF), shoot tolerance index, and root tolerance index (TI) were calculated. Then, a scanning electron microscopy (SEM) was used to observe the stomata in fresh leaves under metal stress. Finally, plant biomass indices such as root and shoot dry weight (DW) were measured.

### Determination of Antioxidant Enzyme Activities

Bamboo leaves (0.5 g) were crushed in a mortar and pestle, ground into powder, mixed into liquid nitrogen, and kept at 2°C–8°C. The obtained powder was dissolved in 2 mg of phosphate buffer (pH 7.8) inside a test tube. The obtained homogenate was centrifuged at 2,000–3,000 *g* for 20 min at 4°C. After centrifugation, the obtained supernatant was used for the measurement of antioxidant activities.

The activity of superoxide dismutase (SOD; E.C. 1.15.1.1) was quantified based on the method of Beauchamp and Fridovich (1971), which was achieved by the photoreduction obtained by nitroblue tetrazolium (NBT). Then, the supernatant (100 μl) was mixed with 50 mM phosphate buffer (pH 7.8), which was added to a solution containing 13 mM methionine, 0.1 mM ethylenediaminetetraacetic acid (EDTA), 75 μM NBT, and 2 μM riboflavin. In the next step, the obtained solution was exposed to fluorescent lamps for 10 min. To determine the SOD, the absorbance was measured at 560 nm by a spectrometer. The activity of catalase (CAT; E.C. 1.11.1.6) was recorded by Urbanek et al. (1991). In this process, 0.1 ml of the extracted sample was dissolved in 3 ml phosphate buffer (pH 6.8) by using extinction coefficient of H_2_O_2_ in 39.4 M^-1^ cm^-1^. CAT activity was recorded by measuring the decline in absorbance at 240 nm. The activity of ascorbate peroxidase (APX; E.C. 1.11.1.11) was determined according to the method of Nakano and Asada (1981). Therefore, in this measurement, the reaction buffer solution including 50 mM potassium phosphate (pH 7.0) was added to the extracted sample, and then 0.1 mM EDTA, 0.1 mM H_2_O_2_, and 0.5 mM ascorbate were added to the solution. Hence, APX antioxidant activity was obtained by recording the reduction in absorbance at 290 nm (the coefficient of absorbance at 2.8 mM^−i^ cm^−1^). The activity of glutathione reductase (GR; E.C. 1.6.4.2) was measured by the method of Foyer and Halliwell (1976) with some modifications. For this index, the mixture consisted of extract samples which were added to 100 mM phosphate buffer (pH 7.8) and then mixed by 3.0 mM oxidized glutathione, 0.05 mM nicotinamide adenine dinucleotide phosphate (NADPH), 0.1 μM EDTA, 50 μl of the enzyme extract, and 1.0 ml NADPH oxidation was recorded in the absorbance of the 340 nm twice. The first time included the addition of H_2_O_2_, and the second time occurred 1 min later. The difference between the two data points was determined by the extinction coefficient of the NADPH molar (6.22 mM cm^−1^). The activity of GR was obtained as U mg^−1^ protein.

### Measurement of Tyrosine Ammonia-Lyase and Phenylalanine Ammonia-Lyase Activity

The activity of TAL was determined by the method of Berner et al. (2006). Therefore, 20 μl of the extracted sample was added to the solution that contained tyrosine (30 mM) and boric acid buffer (pH 8.5; 500 μl). Then, in the final step, the TAL activity was recorded by the absorbance at 310 nm after 30 min. p-Coumaric acid was used for the standard curve. PAL activity was measured according to the method of Berner et al. (2006). Therefore, 500 μl boric acid buffer (pH 8) was mixed in the 20 μl extracted samples, and the absorbance was recorded at 290 nm after 30 min. In this test, various levels of Ecinnamic acid were used as the standard curve.

### Measurement of Hydrogen Peroxide, Lipid Peroxidation, Lipoxygenase Activity, and Electrolyte Leakage

The H_2_O_2_ content was determined by the method of Velikova et al. (2000). For this purpose, 0.1% (w/v) trichloroacetic acid (TCA; 5 ml) was homogenized with leaf samples (0.5 g). Then, it was centrifuged at 12,000*g* for 15 min. In the next step, supernatant (0.5 ml) was added to 10 mM potassium phosphate buffer (pH 7.0; 0.5 ml) and 1 M potassium iodide (1 ml). In final step, the absorbance at 390 nm was recorded. For the calculation of H_2_O_2_, one standard calibration curve was used. The estimation of lipid peroxidation was made by the TBARS content, which is used as a cell peroxidation indicator. In addition, it was used by the method of Cakmak and Horst (1991). In this experiment, 0.5 g of leaf samples were crushed in containers of 0.1% (w/v) TCA (5 ml). As for the next step, the mixture was centrifuged at 12,000*g* for 7 min. The obtained supernatant was added to 4 ml of 0.5% (w/v) TBA and 4 ml of 20% (w/v) TCA and kept at 90°C for 30 min. In the following step, the mixture was centrifuged at 10,000*g* for 5 min. Then, the TBARS content was recorded by absorbance at 532 nm. The TBARS content was determined as μM g^−1^ leaf FW. The lipoxygenase (LOX) activity was determined based on the methods of Grossman and Zakut (1979) and Sekha and Reddy (1982). Using these methods, 25 ml of the 0.1 M sodium tetraborate containing 0.1% Tween 20 was added to 10 μl linoleic acid. Then, 0.1 ml of the solution was added to an optimal amount (2.9 ml) of 0.1 M phosphate buffer of pH 4–5. EL was measured according to the method of Valentovic et al. (2006). Using this method, 0.3 g of leaf sample were added to 15 ml of deionized water. Then, the samples were preserved at 27°C for 2 h. Then, the EC_1_ (electrical conductivity) of the solution was recorded. In the next step, the samples were kept in one autoclaved at 120°C for 17 min. Finally, (EC_2_) electrical conductivity was recorded again. The EC was obtained as follows:


$$\mathrm{EC}\;\left(\%\right)=\left({\mathrm{EC}}_{1}/{\mathrm{EC}}_{2}\right)\times 100$$

### Measurement of Glycine Betaine and Proline Contents

The content of GB was determined by the method of Grieve and Grattan (1983). The samples (leaves) were placed in an oven to dry at a controlled temperature (80°C). The dried samples were finely ground with deionized water at 110°C for 40 min. The GB concentration was recorded at 365 nm, which was obtained by the mixture of dry leaf powder after reaction with KI–I_2_.

The proline content was measured based on the ninhydrin method (Bates et al., 1973). Therefore, 400 mg of the samples (leaf) were homogenized in sulfosalicylic acid. In the next step, 3 ml glacial acetic acid and 3 ml acid ninhydrin were added to the mixture. Then, the solution was heated at 110°C. The supernatant was extracted with toluene, and the free toluene was measured by absorption at 528 nm. In this experiment, the standard used L-proline.

### Determination of Total Phenolics, Flavonols, and Tocopherols

Dry leaf samples (0.5 g) were mixed in 80% methanol (5 ml) and then centrifuged at 7,000*g* for 15 min. Methanolic extract was obtained for use in the experiment. The content of total phenolics was measured by the Akkol method (Akkol et al., 2008). For this purpose, 2.5 ml of 10% Folin–Ciocalteu reagent was added to 0.1 ml of methanolic extract. Then, the obtained mixture was neutralized by 7% sodium bicarbonate. In the final step, the content of total phenolics was recorded by absorbance at 765 nm. The results were expressed by using gallic acid calibration (mg GAE g^−1^ plant material). The flavonol content was determined according to the methods of Akkol (Akkol et al., 2008). In this test, 0.5 ml methanolic extract was homogenized with 1.5 ml of 5% sodium acetate and 2% 0.4 ml aluminum chloride. The obtained supernatant was preserved for 2.5 h at normal room temperature, and then flavonoid content was recorded by absorbance at 445 nm. The calibration for rutin was used for the calculations and the results were expressed as (mg RE/g of plant material). The content of tocopherol was determined by the method of Kayden et al. (1973). In this study, 0.1 g of leaves and samples were added to 3 ml ethanol, and then the mixture was transferred to a centrifuge machine and centrifuged at 10,000*g* for 10 min. Next, the ethanol extract (0.1 ml) was added to 0.2 ml ferric chloride (0.001 M), 0.2 ml bathophe-nanthroline (0.2%), and 0.2 ml phosphoric acid (1 mM). The tocopherol content was obtained by recording the absorbance at 534 nm. TAC was determined by comparison with the tocopherol acetate standard calibration curve. The amount of TAC was expressed for extract samples in mM tocopherol acetate equivalent/g plant material.

### Assay of Chlorophyll and Carotenoid Contents

Chlorophyll pigments including Chl *a*, Chl *b*, total Chl, and carotenoid contents were recorded according to the Lichtenthaler method (Lichtenthaler and Buschmann, 2001). For this study, 0.5 g of bamboo samples (leaf) were exposed to liquid nitrogen in a mortar and then crushed. Then, the obtained powder was mixed with 20 ml of 80% acetone at a temperature of 0°C–5°C. The mixture was extracted and centrifuged at 7,000 × *g* for 15 min. Finally, the obtained supernatant was placed in a UV/vis spectrometer. Therefore, Chl a, Chl b, and carotenoid contents were measured by recording the absorbance at 663, 645, and 470 nm. To obtain the final data, the following formulae were used, which are displayed in units of mg g^−1^ fresh weight:


$$\mathrm{Chl}\;a=12.25A_{663}-2.79A_{647}$$

$$\mathrm{Chl}\;b=21.50A_{647}-5.10A_{663}$$

Total Chl=Chla+Chlb

$$\mathrm{Carotenoid}=1,000A_{470}-1.82\mathrm{Chl}\;a-95.15\;\mathrm{Chl}\;b\mathrm{/225}$$

### Assay of ZnO-NP Contents and As and Hg Accumulation in Leaves, Stems, and Roots of Bamboo Species

The quantities of ZnO-NPs and As and Hg in leaves, stems, and roots of bamboo species were measured in Nanjing Forestry University lab, and the sample preparations were according to the method of Karimi et al. (2013) with some modification. The different plant organs, including leaves, stems, and roots, were carefully washed and then dried at an oven temperature of 110°C for 5–8 h. Then, nitric acid (70%) was added to the samples at a temperature of 80°C for 15 min. Then, this process was continued by centrifugation of the samples at 10,000*g* for 7 min. For the determination of ZnO-NP contents and As and Hg accumulation in leaves, stems, and roots, the recordings were performed by atomic absorption spectrometry (AAS), which was equipped with a Zeeman-effect background correction system and a graphite furnace (Analyst 800, Perkin Elmer). These instruments were used to determine the metal accumulation through analysis. Determination of metal standards was conducted based on nitric acid (2.5%) using a spectral scan. The machine calibration of the standard (Perkin Elmer), a standard including all of the elements in an inorganic target analyst list (TAL), was run at optimum intervals in an unattended automatic analysis run mode.

### Determination of Plant Biomass

In the last step of the experiment, bamboo samples were separated from MS medium and divided into two types of roots and shoot samples. For the analysis of physiobiochemical treatments in the future, a tiny fraction of the fresh shoots was stored under cold conditions at −80°C. The other parts of the plant root and plant shoot were placed in an oven to dry at the optimum temperature of 70°C for 48 h. Then, the plant biomass, including shoot dry weight (SHDW) and root dry weight (RDW), were obtained after weighing the samples.

### Translocation Factor and Tolerance Index Assays

To determine the performance of various concentrations of ZnO-NPs in coping with metal toxicity and to identify the involved mechanisms, the TF and TI were obtained. This was calculated based on the Souri method (Souri et al., 2020). The value was calculated by the following formulae:


Translocation factorTF=the concentrations ofZnO-NPsand metal/metalloidAsandHgin the plant shootsleavesμgg−1/the concentrations ofZnO-NPsand metal/metalloidAsandHgin the plant rootsμgg−1

Tolerance indexTIof shoots=dryweightDWof plant shoots fromZnO-NPand metal/metalloidAsandHgtreatmentsg/dryweightDWof the plant shoots from the controlg

Tolerance index(TI)of root=dryweight(DW)of plant root fromZnO-NPsand metal/metalloid(AsandHg)treatment(g)/dryweight(DW)of the plant root from control(g)

### Scanning Electron Microscopy

The bamboo leaves were observed based on method of Li et al. (2016b) with some corrections and modifications. The central section of fresh leaves of the bamboo species was used for SEM. For this purpose, after the leaves were cut into 6.0 mm × 6.0 mm parts, the pieces were transferred to an oven and dried at 70°C for 20 min. A gold anion sputter apparatus (Model E-1010 Hitachi Ion Sputter JEOL, Japan) operated at 16 mA for 60 s was used to coat the samples. The middle section of leaves was scanned using a scanning electron microscope (SEM) system (JSM-6380, JEOL, Tokyo, Japan) operated at a voltage of 15–25 kV.

### Statistical Analysis

Data were analyzed by a two-way factorial design with four replicates. ANOVA was conducted using the R statistical software package. Tukey’s test was used for comparison of the mean differences between treatments at the *p* < 0.05 probability level.

## Results

### ZnO-NPs Increase the Antioxidant Activity Under As and Hg Toxicity

**Figure 1:**
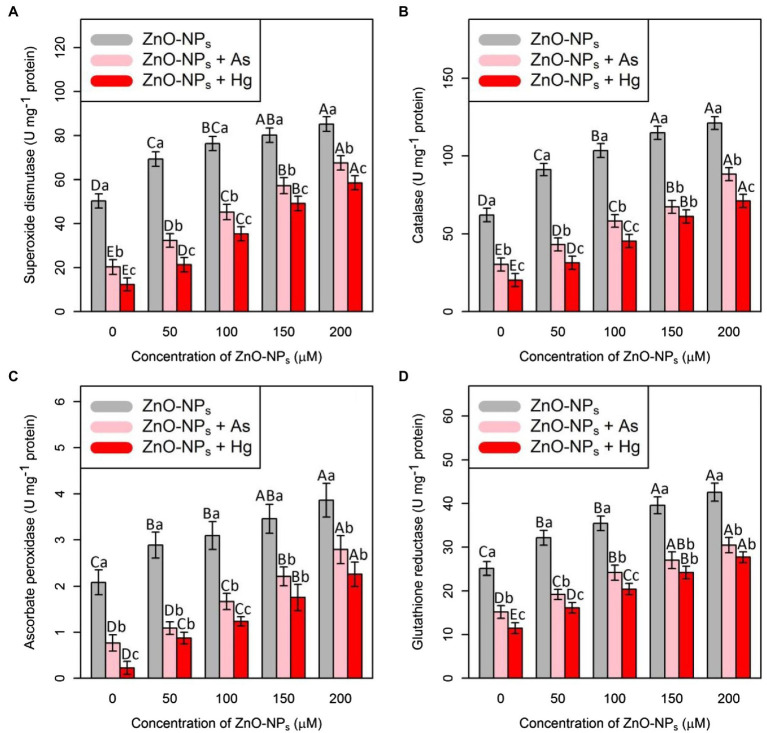
The impact of zinc oxide nanoparticles (ZnO-NPs) concentrations on the antioxidant enzyme activities **(A)** superoxide dismutase (SOD), **(B)** catalase (CAT), **(C)** ascorbate peroxidase (APX), and**(D)** glutathione reductase (GR) in bamboo species (*Pleioblastus pygmaeus*) with 150 μM As and 150 μM Hg. The capital letters indicated statistically significant differences across various concentration of ZnO-NPs alone or in combination with 150 μM As and 150 μM Hg (the bars with the same colors), while the lowercase letters indicated statistically significant differences within each level of ZnO-NPs alone or in combination with 150 μM As and 150 μM Hg (the bars with different colors) according to Tukey′s test (*p* < 0.05).

The ANOVA showed that there were significant differences between the various levels of ZnO-NPs alone and in combination with As and Hg and antioxidant activities (SOD, CAT, APX, and GR; *p* < 0.001). Therefore, ZnO-NPs could increase antioxidant enzyme activity under As and Hg (Figure 1). The greatest increase in antioxidant activity was related to high levels of ZnO-NPs (200 and 150 μM), with 69%, and 59% increases in SOD activity, 95%, and 85% increases in CAT activity, 84%, and 65% increases in APX activity and 69%, and 57% increases in GR activity in comparison with their control treatments, respectively. Additionally, the results indicated that the treatments with 150 μM As and 150 μM Hg produced the lowest stimulation of the antioxidants, as shown by the SOD, CAT, APX, and GR activities, which were reduced by 59, 52, 63, and 40% by As and 75, 68, 89, and 46% by Hg, respectively, compared with their control.

### ZnO-NPs Increase Tyrosine Ammonia-Lyase Activity and Phenylalanine Ammonia-Lyase Activity Under As and Hg Toxicity

**Figure 2:**
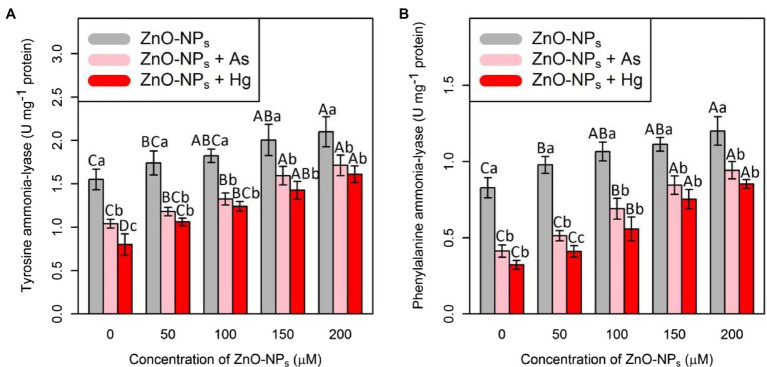
The impact of ZnO-NPs concentrations on **(A)** tyrosine ammonia-lyase (TAL) activity and **(B)** phenylalanine ammonia-lyase (PAL) in bamboo species (*Pleioblastus pygmaeus*) with 150 μM As and 150 μM Hg. The capital letters indicated statistically significant differences across various concentration of ZnO-NPs alone or in combination with 150 μM As and 150 μM Hg (the bars with the same colors), while the lowercase letters indicated statistically significant differences within each level of ZnO-NPs alone or in combination with 150 μM As and 150 μM Hg (the bars with different colors) according to Tukey’s test (*p* < 0.05).

Tyrosine ammonia-lyase and PAL are two important antioxidants that were measured in this study. Therefore, the results showed that the addition of ZnO-NPs alone and in combination with 150 μM As and 150 μM Hg significantly increased both TAL and PAL activities (*p* < 0.001; Figure 2). In this study, the greatest increase in TAL and PAL activities was related to 200 μM ZnO-NPs, with 35%, and 46% increases in TAL and PAL activities, respectively, compared with their control treatment. The lowest one was related to a concentration of 150 μM As and 150 μM Hg with 32%, and 48% reduction in TAL activities, and 50 and 60% reductions in PAL activities in comparison with control treatments. Therefore, we suggested that ZnO-NPs have the ability to increase TAL and PAL activities under metal/metalloid stress (Figure 2).

### ZnO-NPs Have a Positive Impact on the Reduction of H_2_O_2_ Content, Lipid Peroxidation, and Electrolyte Leakage Under As and Hg

**Figure 3:**
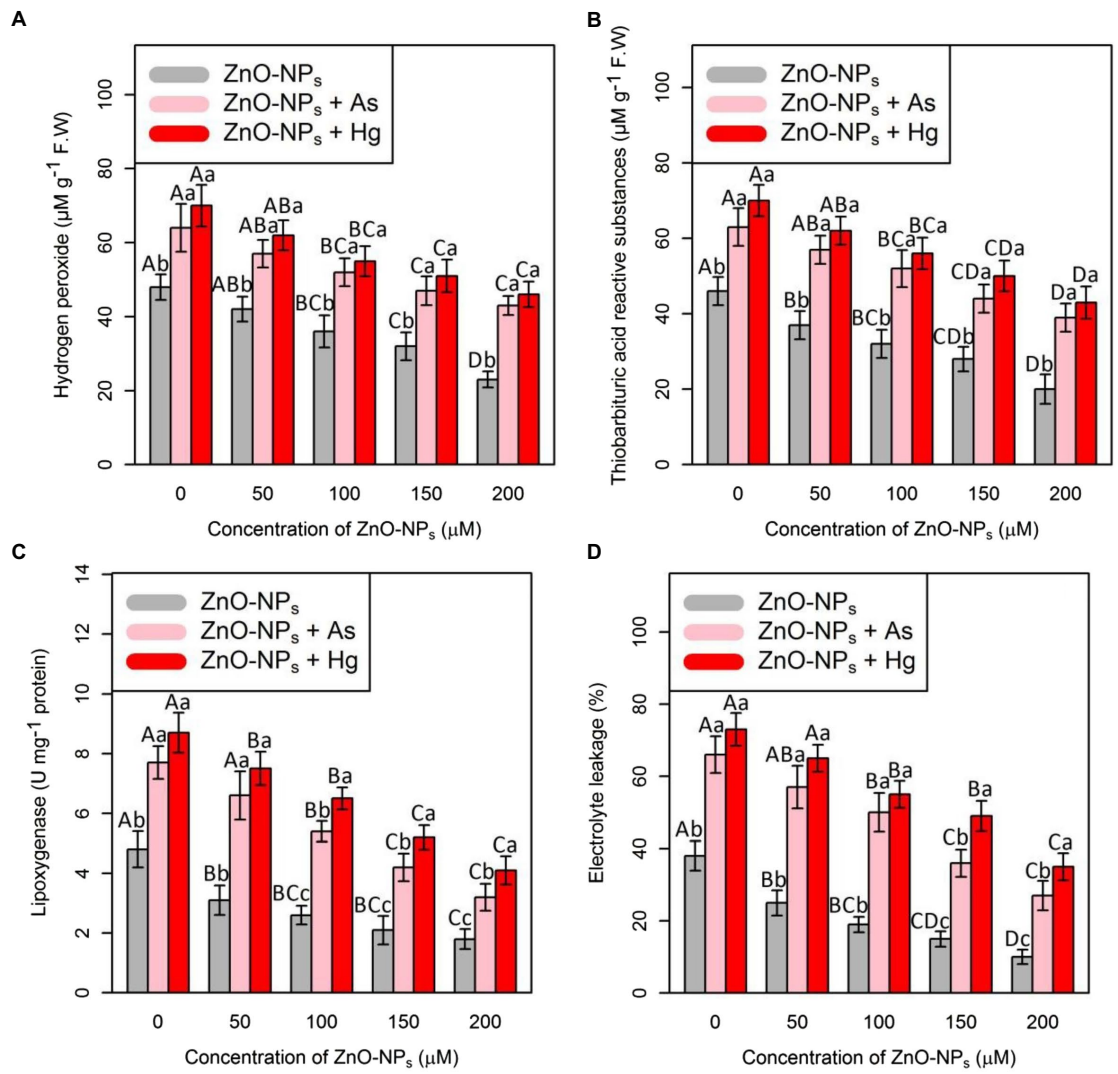
The impact of ZnO-NPs concentrations on **(A)** hydrogen peroxide (H_2_O_2_) content, **(B)** thiobarbituric acid reactive substances (TBARS), **(C)** lipoxygenase activity (LOX), and **(D)** electrolyte leakage (EL) in bamboo species (*Pleioblastus pygmaeus*) with 150 μM As and 150 μM Hg. The capital letters indicated statistically significant differences across various concentration of ZnO-NPs alone or in combination with 150 μM As and 150 μM Hg (the bars with the same colors), while the lowercase letters indicated statistically significant differences within each level of ZnO-NPs alone or in combination with 150 μM As and 150 μM Hg (the bars with different colors) according to Tukey’s test (*p* < 0.05).

The investigation of the effect of HMs on plant membrane and cell peroxidation is important. For estimation, the impact of ZnO-NPs on ROS compounds and lipid peroxidation and the rate of plant membrane injury, H_2_O_2_ contents, TBARS, LOX, and EL in bamboo species were measured. The results showed that there was a significant difference between the various levels of ZnO-NPs alone and in the form of combinations with As and Hg metals (*p* < 0.001; Figure 3). Therefore, the results revealed that ZnO-NPs can reduce oxidative stress and membrane injury caused by As and Hg (Figure 3). In this study, the greatest reduction in H_2_O_2_ content, TBARS and LOX activity, as well as EL percentage, was related to 200 μM ZnO-NPs with 52, 56, 62, and 73% reductions compared with their control treatments. This showed the ability of ZnO-NPs to reduce membrane injury. Therefore, the combination of ZnO-NPs with metal/metalloid has a strong ability to reduce oxidative stress in plants, which can be related to the stimulation of antioxidant activities by separate concentrations of ZnO-NPs.

### ZnO-NPs Increase Nonenzymatic Antioxidants to Cope With As and Hg

According to the results, the total phenolic, flavonol, and tocopherol contents reacted with an increasing trend upon the addition of ZnO-NPs. Therefore, there was a significant difference between the various levels of ZnO-NPs alone and in the form of combinations with As and Hg (*p* < 0.001; Table 1). In this study, the greatest enhancement of total phenol, flavonol, and tocopherol contents was related to 200 and 150 μM ZnO-NPs with 81, and 73% increases in total phenol and 37%, and 27% increases in flavonol and 44%, and 38% increases in tocopherol content in comparison with their control treatments. According to the results, the addition of ZnO-NPs could significantly increase nonenzyme antioxidants in bamboo plants, which can help to reduce ROS compounds and oxidative stress in plants. On the other hand, the total phenolic, flavonol, and tocopherol contents showed 29, 42, and 36% reductions by 150 μM As and 40, 59, and 54% reductions by 150 μM Hg relative to their control, respectively. This shows the toxic role of metals in reducing nonenzyme activities in the present study.

**Table 1:** Impact of the combination of ZnO-NPs with 150 μM As and 150 μM Hg on the non-enzymatic antioxidants (total phenolics, flavonols, and tocopherols), proline content and glycine betaine (GB) content.

ZnO NPs levels (μM)	As/Hg (μM)	Flavonols (mg RE g^−1^ F.w.)	Tocopherols (mM TAE g^−1^ F.w.)	Total Phenolics (mg GAE g^−1^ F.w.)	Proline (μg g^−1^ F.w.)	GB (μg g^−1^ F.w.)
0	0	270 ± 12.24^Da^	622.50 ± 33.04^Ca^	525.0 ± 45.09^Ca^	320 ± 25.81^Ba^	770.25 ± 34.47^Ca^
0	150 μM As	156.50 ± 12.66^Eb^	392.25 ± 24.70^Eb^	182.50 ± 42.72^Db^	170 ± 16.32^Cb^	460 ± 39.15^Eb^
0	150 μM Hg	110.5 ± 13.20^Ec^	281.25 ± 22.86^Dc^	72.50 ± 45.00^Dc^	130 ± 18.25^Db^	350 ± 29.43^Ec^
50 μM	0	303.75 ± 12.20^Ca^	782.75 ± 50.24^Ba^	742.5 ± 70.88^Ba^	430 ± 31.62^Aa^	990.00 ± 52.91^Ba^
50 μM	150 μM As	191.25 ± 11.44^Db^	472.50 ± 23.90^Db^	267.50 ± 26.29^Db^	230 ± 21.60B^Cb^	620 ± 37.41^Db^
50 μM	150 μM Hg	160.5 ± 12.71^Dc^	423.00 ± 20.81^Cb^	185.00 ± 34.15^Cb^	180 ± 25.81C^Db^	520 ± 37.41^Dc^
100 μM	0	331.50 ± 12.66^BCa^	816.50 ± 58.22^Aba^	800.0 ± 79.58^ABa^	450 ± 33.66^Aa^	1050.25 ± 81.65^ABa^
100 μM	150 μM As	231.75 ± 13.54^Cb^	560.00 ± 33.50^Cb^	447.50 ± 49.91^Cb^	270 ± 25.81^Bb^	730 ± 35.59^Cb^
100 μM	150 μM Hg	201 ± 11.86^Cc^	480.25 ± 32.85^Cb^	287.50 ± 26.29^Bc^	240 ± 29.43B^Cb^	640 ± 39.15^Cb^
150 μM	0	344 ± 14.30^ABa^	862.50 ± 49.24^ABa^	860.0 ± 87.55^ABa^	480 ± 38.29^Aa^	1110.0 ± 82.46^ABa^
150 μM	150 μM As	270.25 ± 14.63^Bb^	652.50 ± 33.04^Bb^	572.50 ± 53.77^Bb^	360 ± 29.43^Ab^	850 ± 65.82^Bb^
150 μM	150 μM Hg	241.5 ± 13.47^Bc^	581.50 ± 29.99^Bb^	492.50 ± 41.93^Ab^	280 ± 34.64^Bc^	750 ± 41.63^Bb^
200 μM	0	371 ± 13.03^Aa^	889.00 ± 68.31^Aa^	898.0 ± 52.59^Aa^	500 ± 41.63^Aa^	1200.00 ± 109.84^Aa^
200 μM	150 μM As	301.75 ± 12.68^Ab^	771.00 ± 47.27^Ab^	748.75 ± 28.97^Ab^	420 ± 45.46^Ab^	980 ± 43.96^Ab^
200 μM	150 μM Hg	275.5 ± 11.73^Ac^	675.00 ± 34.15^Ab^	583.75 ± 60.19^Ac^	370 ± 27.08^Ab^	870 ± 34.64^Ab^

Each data point is the mean ± SE of four replicates. The treatments included four levels of ZnO-NPs (50, 100, 150, and 200 μM) alone and in combination with 150 μM As and 150 μM Hg. The capital letters indicated statistically significant differences across various levels of ZnO-NPs alone or in combination with 150 μM As and 150 μM Hg, while the lowercase letters displayed statistically significant differences within each level of ZnO-NPs alone and in combination with 150 μM As and 150 μM Hg based on Tukey’s test (*p* < 0.05). They are superscripted on top of the numbers.

### ZnO-NPs Increased Proline and Glycine Betaine Content in Bamboo Species Under As and Hg Stress

Previously, it had been reported that Pro and GB can be suitable indicators to measure metal toxicity and plant defense mechanisms’ abilities to respond to stress conditions. The results showed that ZnO-NPs significantly increased GB and Pro (*p* < 0.001), which indicates the role of ZnO-NPs in the reduction of As and Hg (Table 1). According to the obtained results, the greatest increase in Pro and GB was related to 200 μM ZnO-NPs, with 56%, and 55% increases relative to the control treatments, respectively. The lowest amount was related to 150 μM As and 150 μM Hg, with 46 and 59% reductions in Pro and 40 and 54% reductions in GB in comparison with their control treatment.

### ZnO-NPs Improve Chlorophyll and Carotenoid Contents in Bamboo Species Under As and Hg Stress

**Figure 4:**
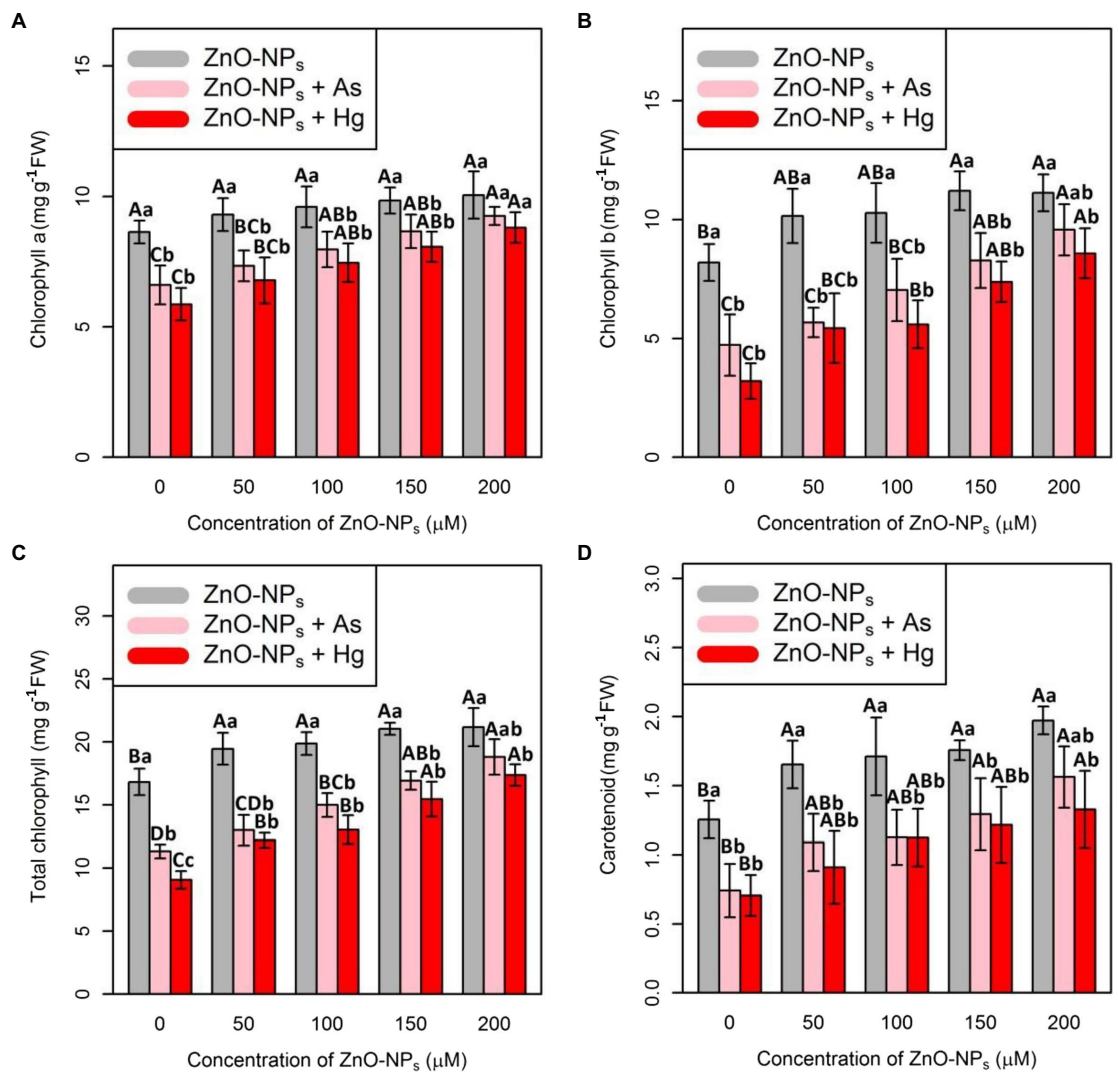
Impact of the combination of ZnO-NPs with 150 μM As and 150 μM Hg on chlorophyll *a* **(A)**, chlorophyll *b* **(B)**, and total chlorophyll **(C)** as well as carotenoid contents **(D)**. Each data point is the mean ± SE of four replicates. The treatments included four levels of ZnO-NPs (50, 100, 150, and 200 μM) alone and in combination with 150 μM As and 150 μM Hg. The capital letters indicated statistically significant differences across various levels of ZnO-NPs alone or in combination with 150 μM As and 150 μM Hg, while the lowercase letters displayed statistically significant differences within each level of ZnO-NPs alone and in combination with 150 μM As and 150 μM Hg based on Tukey’s test (*p* < 0.05). They are superscripted on top of the numbers.

To evaluate the impact of ZnO-NPs on plant photosynthesis and plant metabolism after exposure to As and Hg toxicity, Chl indices, including Chl *a*, Chl *b*, and total Chl as well as carotenoid contents, were measured. In this study, the data recorded a positive impact of various levels of ZnO-NPs on the quantity of Chl indices and carotenoid contents (Figure 4), which demonstrated a significant difference between the various levels of ZnO-NPs alone or in the form of combinations of As and Hg (*p* < 0.001). Therefore, the greatest enhancement of Chl *a*, Chl *b*, total Chl, and carotenoids was related to 200 μM ZnO-NPs with 16, 35, 25, and 57% increases compared with their control treatments, respectively. As expected, 150 μM As and 150 μM Hg had a deleterious impact on Chl and carotenoid indices, which reduced Chl *a*, Chl *b*, total Chl, and carotenoid contents by 23, 42, 32, and 59% by As and 32, 60, 46, and 43% reduction by Hg in comparison with their controls, respectively.

### ZnO-NPs Lead to Increased Plant Growth and Biomass Indices in Bamboo Species Under As and Hg Toxicity

**Figure 5:**
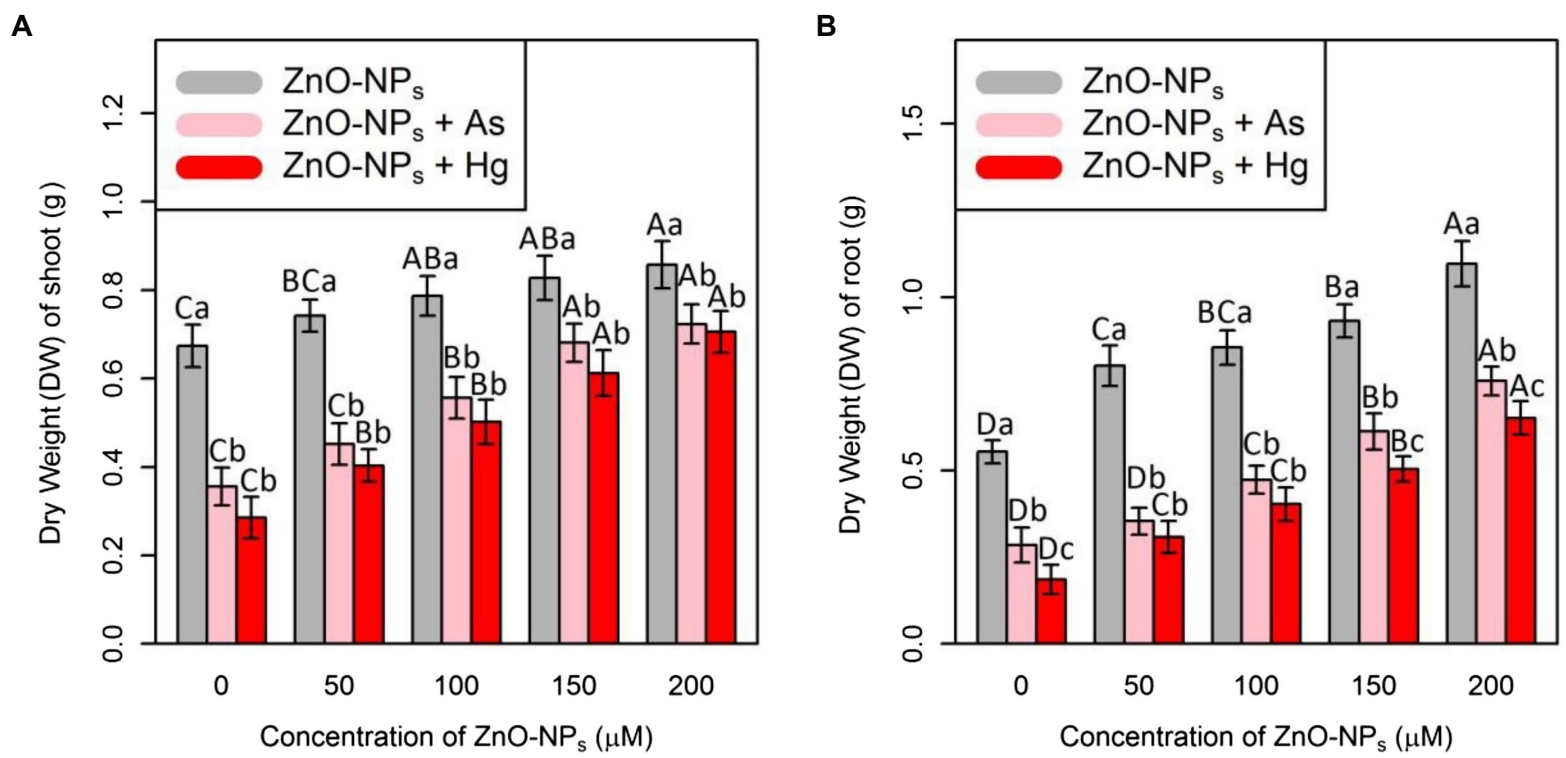
The impact of ZnO-NPs concentrations on **(A)** dry weight (DW) of shoot and **(B)** dry weight (DW) of root in bamboo species (*Pleioblastus pygmaeus*) with 150 μM As and 150 μM Hg. The capital letters indicated statistically significant differences across various concentration of ZnO-NPs alone or in combination with 150 μM As and 150 μM Hg (the bars with the same colors), while the lowercase letters indicated statistically significant differences within each level of ZnO-NPs alone or in combination with 150 μM As and 150 μM Hg (the bars with different colors) according to Tukey’s test (*p* < 0.05).

In this study, plant biomass indices were demonstrated as indicators of plant growth and development, including the bamboo shoot and root dry weight. According to the obtained data analyses, ZnO-NPs significantly increased the dry weight of shoots and roots in bamboo species under two As and Hg. Therefore, the greatest increase in shoots and roots DW was related to the high concentration of ZnO-NPs (150 and 200 μM) with 0.12 and 0.15 g increases in shoots DW and 0.38 and 0.54 g increases in DW of roots in comparison with their control treatments. However, the levels of ZnO-NPs in combination with 100 μM As and 100 μM Hg showed the ability to increase shoots and roots DW in bamboo species (Figure 5; Table 2).

**Table 2:** The percent changes of bamboo biomass in shoot and root dry weight at various levels of ZnO-NPs in combination with 150 μM As and 150 μM Hg compared to those of the control treatment (100%).

ZnO-NPs Levels (μM)	Metal/metalloid levels	Dry shoot weight	Dry root weight
0	150 μM As	47%↓	53%↓
0	150 μM Hg	58%↓	66%↓
50	0	10%↑	44%↑
50	150 μM As	32%↓	36%↓
50	150 μM Hg	40%↓	44%↓
100	0	16%↑	54%↑
100	150 μM As	17%↓	14%↑
100	150 μM Hg	25%↓	27%↓
150	0	23%↑	68%↑
150	150 μM As	1% ↑	10%↑
150	150 μM Hg	9% ↓	9% ↓
200	0	27% ↑	96%↑
200	150 μM As	7%↑	36%↑
200	150 μM Hg	4%↑	18%↑

### Determination of ZnO-NP Accumulation as Well As and Hg Contents in Bamboo Species

The data analysis obtained by this study demonstrated that there was a significant difference between the various levels of ZnO-NPs alone and in the form of combinations with As and Hg (*p* < 0.001). Therefore, the various levels of ZnO-NPs in combination with 150 μM As and 150 μM Hg could reduce the accumulation of metal/metalloid in plant leaves, stems, and roots. The greatest reduction was related to 200 μM ZnO-NPs in combination with 150 μM As and 150 μM Hg, with 66 and 59% reductions in the leaves, 66 and 61% reductions in the stem, and 23 and 47% reductions in the root in comparison with their control, respectively (Table 3).

**Table 3:** The concentrations of ZnO-NPs, As, and, Hg in bamboo leaves, stems, and roots.

ZnO-NPs level (μmol L^−1^)	As/Hg level (μmol L^−1^)	Corresponding As/Hg concentration (μg g^−1^)			Corresponding ZnO-NPs concentration (μg g^−1^)		
		Leaf	Stem	Root	Leaf	Stem	Root
0	0	nd	nd	nd	nd	nd	nd
0	150 μM As	18.65 ± 0.98^Aa^	22.70 ± 0.70^Ab^	15.70 ± 0.94^Cb^	nd	nd	nd
0	150 μM Hg	20.50 ± 1.32^Aa^	24.55 ± 0.94^Aa^	27.85 ± 1.24^Aa^	nd	nd	nd
50 μM	0	nd	nd	nd	13.60 ± 1.10^Ba^	16.92 ± 1.11^Ca^	17.30 ± 1.18^Da^
50 μM	150 μM As	15.35 ± 0.98^Ba^	18.40 ± 0.98^Bb^	22.10 ± 0.97^Ab^	5.22 ± 0.65^Cb^	7.35 ± 0.70^Db^	9.62 ± 0.95^Cb^
50 μM	150 μM Hg	16.80 ± 0.82^Ba^	20.52 ± 0.98^Ba^	24.70 ± 1.21^Ba^	3.30 ± 0.77^Dc^	6.82 ± 0.90^Cb^	7.52 ± 0.77^Cc^
100 μM	0	nd	nd	nd	15.32 ± 1.12^Ba^	18.75 ± 1.27^BCa^	21.45 ± 0.98^Ca^
100 μM	150 μM As	13.60 ± 0.90^Ba^	14.65 ± 1.01^Cb^	19.52 ± 1.23^Bb^	7.37 ± 0.69^Bb^	9.50 ± 0.90^Cb^	10.50 ± 1.06^Cb^
100 μM	150 μM Hg	14.62 ± 0.86^Ca^	16.20 ± 1.12^Ca^	21.70 ± 1.32^Ca^	6.10 ± 0.76^Cb^	8.50 ± 0.65^Cb^	8.35 ± 0.88^Cc^
150 μM	0	nd	nd	nd	17.72 ± 0.95^Aa^	20.55 ± 1.28^ABa^	25.52 ± 1.24^Ba^
150 μM	150 μM As	11.55 ± 0.97^Ca^	11.32 ± 0.99^Db^	15.42 ± 0.98^Cb^	8.50 ± 0.61^Bb^	12.60 ± 1.02^Bb^	13.62 ± 1.10^Bb^
150 μM	150 μM Hg	11.80 ± 0.99^Da^	13.62 ± 1.24^Da^	18.47 ± 1.06^Da^	7.60 ± 0.71^Bb^	10.92 ± 0.87^Bb^	11.50 ± 1.14^Bb^
200 μM	0	nd	nd	nd	19.55 ± 1.25^Aa^	22.42 ± 1.10^Aa^	29.50 ± 1.56^Aa^
200 μM	150 μM As	6.34 ± 0.79^Da^	7.70 ± 0.87^Eb^	11.95 ± 0.75^Db^	10.60 ± 0.94^Ab^	15.80 ± 0.98^Ab^	16.45 ± 1.26^Ab^
200 μM	150 μM Hg	8.30 ± 0.79^Ea^	9.40 ± 1.00^Ea^	14.50 ± 1.32^Ea^	9.72 ± 0.57^Ab^	13.55 ± 0.99^Ac^	14.20 ± 1.09^Ab^

Each data point is the mean ± SE of four replicates. The treatments included four levels of ZnO-NPs (50, 100, 150, and 200 μM) alone and in combination with 150 μM As and 150 μM Hg. The capital letters indicated statistically significant differences across various levels of ZnO-NPs alone or in combination with 150 μM As and 150 μM Hg, while the lowercase letters displayed statistically significant differences within each level of ZnO-NPs alone and in combination with 150 μM As and 150 μM Hg based on Tukey’s test (*p* < 0.05). They are superscripted on top of the numbers.

nd, not detected.

### Determination of the Translocation Factor and Tolerance Index in Bamboo Species Under As and Hg Toxicity

In this study, to investigate the involved mechanisms in the reduction of metal/metalloid by ZnO-NPs, TF, and TI were calculated. According to the data from Table 4, the tested levels of ZnO-NPs did not significantly reduce the TF value for Hg (although a positive downward trend could be observed); in the case of As, there was a clear decrease in translocation in response to the use of ZnO-NPs, but no dose–response correlation was observed (no differences between the different levels of ZnO-NPs). On the other hand, the investigation of the tolerance index in shoots and roots demonstrated that ZnO-NPs remarkably increased plant tolerance in combination with As and Hg, and the results showed that ZnO-NPs in combination with As and Hg increased the tolerance index in bamboo plants by 7 and 4% enhancement in shoots and 37 and 18% enhancement in roots, respectively (Table 4).

**Table 4:** The change in translocation factor (TF) and tolerance index (TI) in shoot and root at different concentrations of ZnO-NPs in combination with 150 μM As and 150 μM Hg compared to those of the control treatment.

ZnO-NPs levels	As/Hg level	Translocation factor (TF)	Tolerance index (TI; shoot)	Tolerance index (TI; root)
0	0	0.00 ± 0.00^Cb^	1.00 ± 0.00^Ca^	1.00 ± 0.00^Ca^
0	150 μM As	1.21 ± 0.02^Aa^	0.53 ± 0.10^Db^	0.51 ± 0.10^Db^
0	150 μM Hg	0.73 ± 0.07^Ac^	0.42 ± 0.09^Db^	0.33 ± 0.06^Dc^
50 μM	0	0.78 ± 0.05^Aa^	1.09 ± 0.05^BCa^	1.45 ± 0.19^Ba^
50 μM	150 μM As	0.64 ± 0.04^Bb^	0.67 ± 0.07^CDb^	0.63 ± 0.05^CDb^
50 μM	150 μM Hg	0.62 ± 0.04^Ab^	0.60 ± 0.09^CDb^	0.55 ± 0.11^CDb^
100 μM	0	0.71 ± 0.08^ABa^	1.16 ± 0.07^ABa^	1.54 ± 0.18^Ba^
100 μM	150 μM As	0.70 ± 0.08^Ba^	0.82 ± 0.10^BCb^	0.85 ± 0.12^BCb^
100 μM	150 μM Hg	0.69 ± 0.05^Aa^	0.74 ± 0.10^BCb^	0.73 ± 0.13^BCb^
150 μM	0	0.69 ± 0.06^ABa^	1.22 ± 0.08^ABa^	1.68 ± 0.16^ABa^
150 μM	150 μM As	0.69 ± 0.02^Ba^	1.01 ± 0.07^ABb^	1.10 ± 0.16^ABb^
150 μM	150 μM Hg	0.64 ± 0.06^Aa^	0.91 ± 0.11^ABb^	0.91 ± 0.12^Bb^
200 μM	0	0.66 ± 0.00^Ba^	1.27 ± 0.10^Aa^	1.97 ± 0.13^Aa^
200 μM	150 μM As	0.59 ± 0.04^Bb^	1.07 ± 0.13^Aab^	1.37 ± 0.16^Ab^
200 μM	150 μM Hg	0.62 ± 0.02^Aab^	1.04 ± 0.06^Ab^	1.18 ± 0.15^Ab^

Each data point is the mean ± SE of four replicates. The treatments included four levels of ZnO-NPs (50, 100, 150, and 200 μM) alone and in combination with 150 μM As and 150 μM Hg. The capital letters indicated statistically significant differences across various levels of ZnO-NPs alone or in combination with 150 μM As and 150 μM Hg, while the lowercase letters displayed statistically significant differences within each level of ZnO-NPs alone and in combination with 150 μM As and 150 μM Hg based on Tukey’s test (*p* < 0.05). They are superscripted on top of the numbers.

### Impact of ZnO-NPs on Stomatal Closure in the Leaf Epidermis

**Figure 6:**
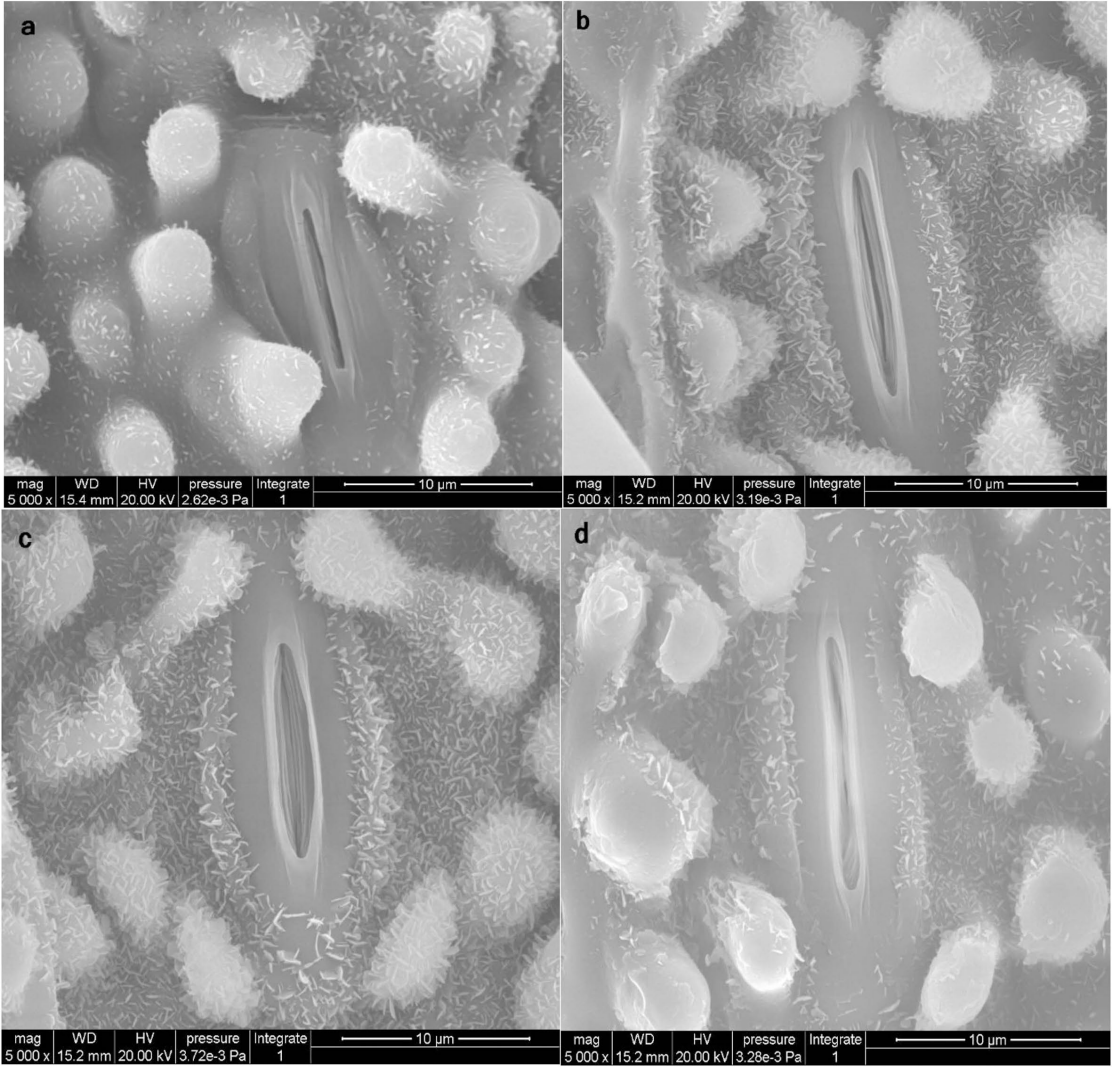
Scanning electron microscopy (SEM) images of stomata closure (opening and closing of stomata) on the leaf surface of bamboo (*Pleioblastus pygmaea*). Image **(a)** displays the control treatment under normal conditions. Image **(b)** displays the condition of stomata in plants receiving 200 μM ZnO-NPs treatment. Images **(c,d)** display the condition of stomata receiving 150 μM As and 150 μM Hg (**c** = As, **d** = Hg) treatment in combination with 200 μM ZnO-NPs. The results suggest that ZnO-NPs kept stomata open when combined with 150 μM As and can also help maintain stomata in a semi open and closed state when combined with 150 μM Hg.

As shown in Table 3, Zn in the form of ZnO-NPs reduced As and Hg accumulation, with alterations in metal/metalloid translocation from the roots to the shoots of bamboo. This phenomenon impacted the opening and closing of stomata in the leaves. Thus, ZnO-NPs could regulate the opening and closing of stomata in plants under As and Hg, which might be attributed to an enhancement in the antioxidant capacity in cells (Figure 6). We suggest that optimal levels of ZnO-NPs may regulate the function of stomatal pores *via* certain mechanisms, such as electrochemical and hydraulic adjustments in guard cells and osmotic pressure regulation. These processes may be related to the ability of ZnO-NPs to scavenge H_2_O_2_ and reduce the extent of lipid peroxidation by increasing the antioxidant capacity.

## Discussion

Nanoparticles can absorb and translocate HMs through their special surface area (Liu et al., 2020). Among them, Zn, in the form of ZnO-NPs, is used as a micronutrient that can help plant growth and development (Kloubert and Rink, 2015). ZnO-NPs can remove HMs from the surface with the exchange of HM ions in water solutions to improve plant growth (Venkatachalam et al., 2017a). It has been shown that excess HMs in plants lead to the generation of ROS compounds and oxidative stress in the plant (Gechev and Petrov, 2020; Wani et al., 2021), which cause damage that includes wall thickness, osmotic shock or stress, decreased water potential and transpiration, lipid membrane, protein, cell nuclear and DNA injury and, finally, the disturbance of plant photosynthesis, and plant growth and development (Akhtar et al., 2021). Therefore, plants adopt a different strategy in the form of antioxidant defense to scavenge ROS compounds (Emamverdian et al., 2018). Antioxidants can ameliorate the toxicity of H_2_O_2_ by converting it into oxygen and water (Jiang et al., 2021). However, as reported in many studies, the efficiency of the antioxidant system depends on the severity, exposure time, and type of stress as well as plant species (Dawood et al., 2012; Hasanuzzaman et al., 2013, 2019). For instance, in a study on rice (*Oryza sativa*), the results showed that CAT activity decreased in the plants under aluminum (Al) toxicity due to the inhibited enzymes subunit assembly, leading to a significant reduction in enzyme synthesis (Sharma and Dubey, 2007). This shows that the severity of oxidative stress can have a negative impact on the synthesis of enzymes and diminish their functions. Our results demonstrated that ZnO-NPs can increase antioxidant activity, especially SOD and CAT, under metal toxicity, which has been evidentially confirmed in *Triticum aestivum* (Hussain et al., 2018), *Zea mays* (Rizwan et al., 2019a,b), and rice (Faizan et al., 2021b). SOD is the front line of the antioxidant defense system, converting O_2_– to H_2_O_2_ with less toxicity, and CAT has completed this process by scavenging H_2_O_2_ to H_2_O and O_2_– (Venkatachalam et al., 2017b). Therefore, they play an essential role in the scavenging of ROS; a process that the present study suggests could be increased. However, all antioxidant activities (SOD, CAT, GR, and APX) were increased following the addition of ZnO-NPs under 150 μM As and 150 μM Hg.

Tyrosine ammonia-lyase and phenylalanine ammonia-lyase (PAL) are two key enzymes in phenolics compounds and biosynthesis pathways. Phenolics compounds include nonenzymatic antioxidants that are involved in ROS scavenging (Rezayian et al., 2020). According to our results, while 150 μM As and 150 μM Hg reduced the TAL and PAL activities, the addition of ZnO-NPs to metal concentrations increased TAL and PAL activities in bamboo species, which demonstrated the role of ZnO-NPs in the stimulation of phenolics content HMs. Therefore, the total phenolics, flavonols, and tocopherols contents were accounted for. Hence, in this study, the results showed that different concentrations of ZnO-NPs increased the phenolics content, including total phenolics, flavonols, and tocopherols, in bamboo species under metal stress. An increase in phenolics content has been reported in the previous studies (García-López et al., 2018; Rezayian et al., 2020). Therefore, we suggest that the combination of ZnO-NPs with increasing antioxidant enzymes capacities improves the plant defense mechanism under ROS conditions.

Many studies have reported that HMs lead to the generation of ROS compounds (H_2_O_2_ and O_2_–; Ahmad et al., 2019; Bhat et al., 2019; Kaya et al., 2020), which finally leads to plant death caused by oxidative stress. However, the results obtained in our study indicated that ZnO-NPs reduced the accumulation and content of H_2_O_2_ and TBARS in the plants under As and Hg toxicity. A similar result was reported by other authors (Li et al., 2016a; Faizan et al., 2020, 2021a,b). Our study demonstrated the role of ZnO-NPs in the activation of plant antioxidant capacities. On the other hand, ZnO-NPs with adsorption mechanisms enhance the interaction between metal ions and NPs to scavenge ROS compounds in plants under metal stress (Maity et al., 2018).

Zinc can play an essential role in the biological regulation of the cell membrane *via* binding groups of sulfhydryl with phospholipids under critical situations such as stress conditions (Hafeez et al., 2013). In this study, LOX showed increasing lipid peroxidation with one important role in the oxidation of poly-unsaturated fatty acids. EL acts as an indicator of cell membrane damage. Our results showed that all concentrations of ZnO-NPs could reduce the EL and LOX contents under 150 μM As and 150 μM Hg. However, the high concentration of ZnO-NPs had the most impact on the reduction of EL and LOX. Therefore, we suggest that ZnO-NPs can protect the cell membrane under metal/metalloid toxicity, which is related to scavenging ROS compounds and improving the plant antioxidant capacity.

Proline and GB are two syntheses of osmolytes that can preserve plant cells from dehydration stress (Ahmad et al., 2019). Pro accumulation in plants plays an important role in plant growth regulation under stress conditions (Torabian et al., 2016), and the positive role of Pro has been revealed in ROS scavenging, membrane stabilization, and finally osmotic stress (Bandurska, 2001). In this study, our results showed that ZnO-NPs can increase the accumulation of Pro in bamboo species, which is related to the role of ZnO-NPs in the expression of genes involved in Pro biosynthesis (Faizan et al., 2021a,b). This result is confirmed by the findings of other studies (Helaly et al., 2014; Faizan et al., 2020). GB acts as an important osmoregulator in plants and can occur at various concentrations in different plant species. It can play a key role in the activation of glutathione (GSH), ascorbic acid (AsA), and glutathione reductase (GR) under HM stress (Ali et al., 2020). Our results showed that ZnO-NPs could increase the accumulation of GB in plants under As and Hg.

It has been reported that ZnO-NPs are involved in the activation of the enzymatic activity of Rubisco and carbonic anhydrase (CA). Therefore, these enzymes, through the expression of particular genes, can increase the chemical energy in the photosynthetic system, which can improve plant photosynthetic properties (Rico et al., 2015). On the other hand, it has been reported that most GBs accumulate in chloroplasts, which can have a positive impact on the effectiveness of safeguarding in photosystem II (PSII) as well as the photosynthesis system in plants under stress. The enhancement in the content of Chl can be an indicator of photosynthesis performance in plants (Altaf et al., 2020). Our results indicated that ZnO-NPs increased the Chl content and carotenoids under 150 μM As and 150 μM Hg in our bamboo species. Therefore, the accumulation of GB can be one of the reasons for increasing Chl and carotenoids contents in plants under stress. This can also involve the activation of antioxidant enzyme activity, including SOD and CAT (Rasheed et al., 2017). Faizan reported that ZnO-NPs enhance Chl contents with a reduction in Cu toxicity in tomato and Cd toxicity in rice (Faizan et al., 2021a,b). This is consistent with the results obtained in the present study. Therefore, it can be suggested that ZnO-NPs can increase photosynthetic properties in plants under As and Hg.

According to SEM observation (Figure 6), our results revealed that ZnO-NPs could regulate stomatal aperture under the stress. So that ZnO-NPs were able to keep stomata open under 150 μM As and also maintained stomata in a semi-open and closed state under 150 μM Hg. Some mechanisms are proposed to explain the stomatal responses to the application of NPs under metal/metalloid stress. Of which, the removal of the As and Hg by NPs through the adsorption process is notable (Fu and Wang, 2011; Burakov et al., 2018) where Zn NPs dispersed on the surface of the leaves and the epidermis can sequester metal ions through adsorption. This has also been shown with different HMs, such as Cu (Mobasherpour et al., 2012), Cd, and tin (Sn; Ghahremani et al., 2017) using different types of nanoparticles. On the other hand, it is reported that ZnO-NPs could be accumulated below the stomata and consequently be transferred through the apoplast pathway. During this transfer, a fraction of the ZnO-NPs releases Zn cations through the dissolution in the apoplast, which could be absorbed by cells of mesophyll and distributed in the lower and upper mesophyll tissues (Zhu et al., 2020) thus the stomatal opening is regulated as revealed in Figure 6.

**Figure 7:**
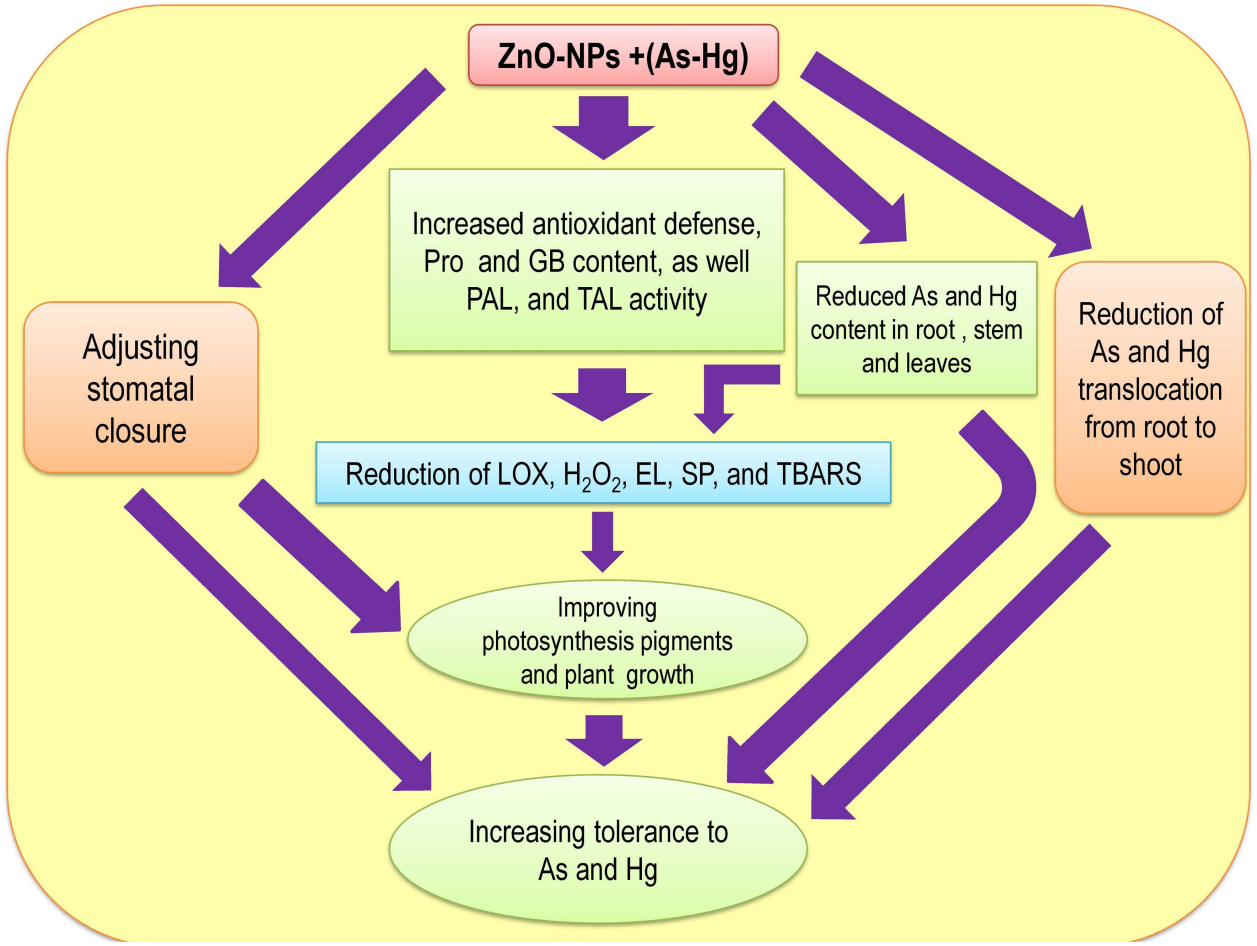
The Mechanistic diagram to indicate the actual mechanisms of ZnO NPs under metal/metalloids toxicity in bamboo.

Many studies have reported that ZnO-NPs reduce HM accumulation in plants (Skiba et al., 2020; Faizan et al., 2021a,b), which is related to the fact that ZnO-NPs precipitate the HM content on the surface of roots. This limits the absorption and translocation of HMs from roots to aerial organs (Venkatachalam et al., 2017b; Ahmad et al., 2019). In fact, Zn can be taken up by plant roots quickly and reduce the uptake of HMs by roots (Hasan et al., 2008). Alternatively, Zn can act as a physical barrier that prevents the translocation of metals from roots to shoots, which occurred in our study. Therefore, Zn accumulation in roots limits metal translocation from roots to shoots and reduces metal accumulation in shoots. Hence, the levels of toxic metal/metalloid in the shoots and stems were significantly lower than those in the roots in our present study. This result has been suggested by other studies (Vasiliadou and Dordas, 2009; Garg and Kaur, 2013). Our results indicated that ZnO-NPs can significantly reduce toxic metal/metalloid accumulation in plants under As and Hg toxicity, as displayed in Table 3. Therefore, the reduction in accumulation and limitation of toxic metal/metalloid translocation by levels of ZnO-NPs can be important mechanisms in the reduction of As and Hg in our bamboo species. Zn^2+^ in the form of ZnO-NPs is known as an essential micronutrient that can help plant growth and development (Liu et al., 2015). Zn also plays a role in the synthesis of auxin (IAA), which can help to improve cell expansion and cell division in plants (Begum et al., 2016). The results demonstrated that while 150 μM As and 150 μM Hg reduced the plant biomass (DW of shoots and roots), the addition of different concentrations of ZnO-NPs could help to increase plant biomass under As and Hg. On the other hand, excess HMs in plants lead to reduced cell viability in plant roots, but ZnO-NPs increase the viability of cells in roots. This can help plant growth and development, which has been reported by other researcher (Rajapakse et al., 2017; Faizan et al., 2021a,b). We suggested that the addition of ZnO-NPs increased the plant biomass with the enhancement of plant root and shoot DW. Therefore, this enhancement can be related to improving photosynthesis indices by increasing antioxidant activity and GB accumulation in plants under 150 μM As and 150 μM Hg. There was one major question in this study: Can ZnO-NPs increase plant tolerance under metal/metalloid toxicity? Our results found that ZnO-NPs have the ability to increase TI in plants toxic metal/metalloid toxicity (As and Hg), as shown in Table 4. Therefore, it can be of considerable importance to use bamboo species in phytoremediation technology in the polluted areas. The mechanistic diagram to indicate the actual mechanisms/role of ZnO NPs under metal/metalloid toxicity in bamboo species has been observed in Figure 7 has shown.

## Conclusion

It was concluded that As and Hg had a pernicious impact on the plant growth by increasing ROS compounds, reducing antioxidant activity, injuring the cell membrane, and depressing the plant photosynthesis. The addition of ZnO-NPs increased the plant photosynthesis by boosting antioxidant activities, GB, Pro, PAL, and TAL contents and reducing cell membrane injury *via* a reduction in H_2_O_2_, LOX, TBARS, and EL. Therefore, ZnO-NPs were able to successfully improve the plant biomass and growth under 150 μM As and 150 μM Hg. We suggest that ZnO-NPs can increase bamboo plant tolerance under As and Hg. This enhancement occurred by reducing metal/metalloid accumulation in the plant parts and decreasing toxic metal/metalloid translocation from the roots to the shoots. Consequently, it raises the potential of the bamboo plants to be used in phytoremediation technology in the polluted areas to clean the environment.

## Data Availability Statement

The raw data supporting the conclusions of this article will be made available by the authors, without undue reservation.

## Author Contributions

AE, YD, MH, and JB: conceptualization. AE: statistical analysis. AE, YD, JB, FM, MH, and GL: writing original draft and revised preparation. AE and GL: investigation. AE, YD, and GL: supervision and funding acquisition. AE, YD, and MH: project administration. JB and FM: English editing. All authors contributed to the article and approved the submitted version.

## Funding

This work was supported by the financial support provided by Nanjing Forestry University (Start-Up Research Fund) and Bamboo Research Institute for the current study. Special Fund for this work was supported by Jiangsu Agricultural Science and Technology Innovation Fund, No. CX(18)2031.

## Conflict of Interest

The authors declare that the research was conducted in the absence of any commercial or financial relationships that could be construed as a potential conflict of interest.

## Publisher’s Note

All claims expressed in this article are solely those of the authors and do not necessarily represent those of their affiliated organizations, or those of the publisher, the editors and the reviewers. Any product that may be evaluated in this article, or claim that may be made by its manufacturer, is not guaranteed or endorsed by the publisher.
